# Multi-omics analysis reveals the sensitivity of immunotherapy for unresectable non-small cell lung cancer

**DOI:** 10.3389/fimmu.2025.1479550

**Published:** 2025-02-07

**Authors:** Rui Wu, Kunchen Wei, Xingshuai Huang, Yinge Zhou, Xiao Feng, Xin Dong, Hao Tang

**Affiliations:** ^1^ Key Laboratory of Carcinogenesis and Translational Research (Ministry of Education/Beijing), Department of Thoracic Surgery II, Peking University Cancer Hospital & Institute, Beijing, China; ^2^ Department of Respiratory and Critical Care Medicine, Changzheng Hospital, Navy Medical University, Shanghai, China; ^3^ Department of Anesthesiology, Changzheng Hospital, Navy Medical University, Shanghai, China; ^4^ School of Medicine, Shanghai University, Shanghai, China

**Keywords:** non-small cell lung cancer, immune checkpoint inhibitors, metabolomics, proteomics, prediction models

## Abstract

**Background:**

To construct a prediction model consisting of metabolites and proteins in peripheral blood plasma to predict whether patients with unresectable stage III and IV non-small cell lung cancer can benefit from immunotherapy before it is administered.

**Methods:**

Peripheral blood plasma was collected from unresectable stage III and IV non-small cell lung cancer patients who were negative for driver mutations before receiving immunotherapy. Then we classified samples according to the follow-up results after two courses of immunotherapy and non-targeted metabolomics and proteomics analyses were performed to select different metabolites and proteins. Finally, potential biomarkers were picked out by applying machine learning methods including random forest and stepwise regression and prediction models were constructed by logistic regression.

**Results:**

The presence of metabolites and proteins in peripheral blood plasma was causally associated with both non-small cell lung cancer and PD-L1/PD-1 expression levels. A total of 2 differential metabolites including 5-sulfooxymethylfurfural and Anthranilic acid and 2 differential proteins including Immunoglobulin heavy variable 1-45 and Microfibril-associated glycoprotein 4 were selected as reliable biomarkers. The area under the curve (AUC) of the prediction model built on clinical risks was merely 0.659. The AUC of metabolomics prediction model was 0.977 and the AUC of proteomics was 0.875 while the AUC of the integrative-omics prediction model was 0.955.

**Conclusions:**

Metabolic and protein biomarkers in peripheral blood both have high efficacy and reliability in the prediction of immunotherapy sensitivity in unresectable stage III and IV non-small cell lung cancer, but validation in larger population-based cohorts is still needed.

## Graphical Abstract

The flowchart of the study is presented.

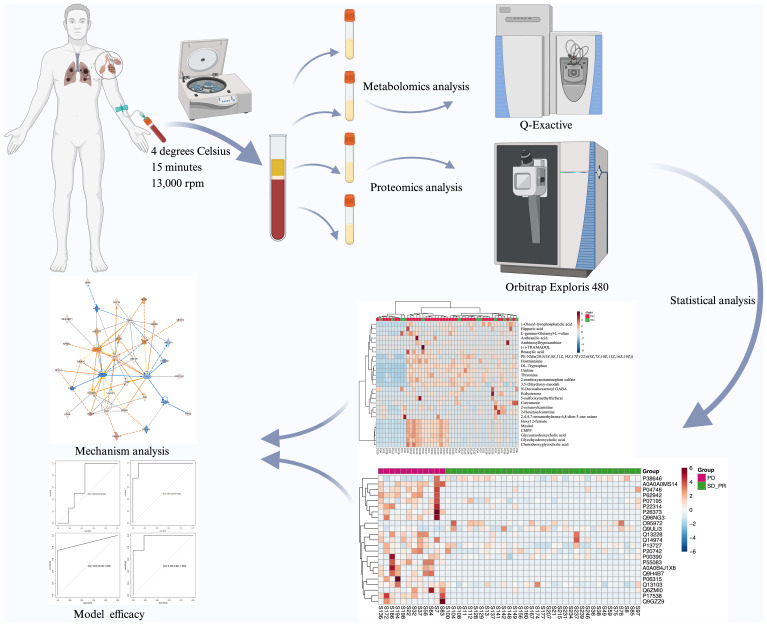

## Background

Lung cancer currently ranks as the second most common malignant tumor globally and the leading cause of cancer-related mortality (1). Pathologically, it can be classified into non-small cell lung cancer (NSCLC) and small cell lung cancer. NSCLC accounts for 80% to 85% of newly diagnosed lung cancer cases. The primary treatment modalities for NSCLC include surgical resection, targeted therapy against tumor-driving genes, conventional chemotherapy, and emerging immune checkpoint inhibitors (2). Approximately 50% of NSCLC patients present with distant organ or lymphatic metastasis at the time of diagnosis (3). For such patients, aside from stage IIIA patient s who may undergo surgical treatment after assessing the efficacy of neoadjuvant therapy, most unresectable stage III and IV NSCLC patients rely on drug therapy as first-line treatment (4). These tumors can be furtherly categorized into NSCLC with driver gene mutations and NSCLC without driver gene mutations. For NSCLC patients with targetable driver gene mutations, the first-line treatment regimen typically consists of targeted therapy against driver genes combined with anti-angiogenic drugs and conventional chemotherapy (5). Conversely, treatment regimens for driver gene-negative patients are stratified based on Eastern Cooperative Oncology Group Performance Status (ECOG) and may include combinations of immune checkpoint inhibitors and conventional chemotherapy or monotherapy with immune checkpoint inhibitors. The advent of immune checkpoint inhibitors, primarily targeting Programmed cell death 1 ligand 1 (PD-L1)/Programmed cell death protein 1 (PD-1), has transformed the treatment landscape for stage III and IV NSCLC (6). For instance, patients treated with pembrolizumab in combination with platinum-based chemotherapy and paclitaxel exhibited an increased overall survival from 10.7 months to 22 months compared with those treated solely with conventional chemotherapy (7). However, it is noteworthy that many patients still do not benefit from immunotherapy and experience tumor progression. Currently, reliable biomarkers and predictive models for identifying patients likely to benefit from immunotherapy before treatment initiation remain lacking.

Metabolomics and proteomics analyses based on human serum samples have provided reliable biomarkers for early diagnosis, treatment plan selection, efficacy monitor and prognosis and helped build many prediction models as biomarkers selection based on peripheral blood has many advantages including low heterogeneity, small trauma and convenience for continuous monitoring (8). As a research tool for comprehensive analysis of changes in endogenous small molecule metabolites occurring in an organism following internal or external stimuli, metabolomics is considered as an extension and endpoint of genomics and proteomics, where small changes in expression levels at the gene and protein level can be amplified at the metabolite level, and thus metabolomics is regarded as a “biochemical phenotype” of the overall functional status of an organism, and is well suited for biomarker screening (9). And as the most downstream of various pathophysiological activities, changes in metabolites also reflect changes in the sensitivity of non-small cell lung cancer to immune checkpoint inhibitor therapy. Proteins are the bearers of various cellular functions, and resolving the spatiotemporal specificity of proteins is a key molecule to understand the heterogeneity of microenvironments and the characteristics of life occurrence and development in tissues and diseases (10). With the promotion of mass spectrometry instrumentation, quantitative histological testing of large-scale samples has begun to gain gradual popularity, and proteomic testing of body fluids, such as plasma, urine, and saliva, for patients with malignant tumors has become an important research method for early diagnosis, pathway discovery, and efficacy monitoring of tumors (11).

In this study, we collected 47 serum samples from unresectable stage III or IV NSCLC patients who received Pembrolizumab treatment and metabolomic and proteomic analyses were performed according to the classification based on follow-up results to explore biomarkers that can be used for early prognosis and provide potential targets for precision therapy (Graph Abstract).

## Patients and methods

### Study design and participants

This study recruited non-small cell lung cancer patients with unresectable stage III or IV driver gene negativity admitted to the Department of Respiratory and Critical Care Medicine of Shanghai Changzheng Hospital from October 2022 to October 2023. The study was approved by the Ethics Committee of Shanghai Changzheng Hospital (2023SL008) and was conducted in accordance with the Declaration of Helsinki. The pathology type of each patient was examined by two experienced pathologists and tumor staging was determined by two experienced radiologists.

5 ml blood from patients who met the inclusion criteria was collected and placed in Ethylene Diamine Tetraacetic Acid (EDTA) blood collection tubes. The tubes were left to stand for 2 hours at 4 degrees Celsius. After centrifugation for 15 minutes at 3,000 rpm and 4 Celsius degrees, the supernatant was taken in a 1.5ml centrifuge tube, labeled with sample information and frozen at -80 degrees Celsius refrigerator for storage. The collection of peripheral blood plasma specimens of the patients was proceeded before immunotherapy, and the efficacy of the immunotherapy was evaluated after patients had received two courses of treatment. Based on the results of the high-resolution chest CT before and after the treatment, the patients were classified into three groups according to the proportion of the reduction of the tumor volume, complete or partial response group, stable disease group, and progressive disease group according to Response Evaluation Criteria in Solid Tumors 1.1 (12). Patients with disappearance of all target lesions or at least a 30% decrease in the sum of diameters of target lesions were grouped into PR/CR, patients who witnessed at least a 20% increase in the sum of diameters of target lesions were grouped into PD group and other patients were grouped into SD group.

### Liquid chromatography-mass spectrometry metabolomics analysis

Thaw serum at 4 degrees Celsius, gently vortex and mix; take 50 microliters of serum in a centrifuge tube; add 200 microliters of internal standard solution; vortex for 1 minute; let stand for 2 minutes; centrifuge at 4 degrees Celsius for 15 minutes at 13,000 rpm; take 100u microliters of supernatant from the wall in the feeder vials and wait for measurement. Take 10 microliters of each supernatant and mix, level and centrifuge for two minutes, immediately take the supernatant into the feed vial as a Quality Control (QC) sample to prevent protein precipitation from floating. The remaining serum was immediately returned to the -80 degrees Celsius refrigerator for refreezing.

A Vanquish ultra-high performance liquid chromatography system, Q-Exactive combination Orbitrap mass spectrometer and C18 column from Thermo Fisher Scientific China Ltd. were used. The temperature was set at 35°C, mobile phase A consisted of 0.1% formic acid and water, and mobile phase B consisted of 0.1% formic acid and acetonitrile at a flow rate of 0.4 ml/min, and the injection volume of each sample was 2 μl, with QC sample inserted in between every eight real samples. The elution gradient was set as 0-2 min: 5-5% (B); 2-13 min: 5-95% (B); 13-15 min: 95 -95% (B); 15-15.1 min: 95-5% (B); 15.1-20 min: 5-5% (B). The ion source for mass spectrometry was a heated electrospray ionization source, HESI, with the voltage set to 3.8 kVt in positive ion mode and 3.2 kVt in negative ion mode, a capillary temperature of 320 degrees Celsius, a mass-to-charge ratio between 100 and 1500, and a shielding gas of 30 liters/minute. After completing the mass spectrometry analysis, the raw data from the Q-Exactive mass spectrometer were exported and proceeded using Thermo Fisher’s Compound Discoverer 3.3 software.

### Statistics and pathway analysis of metabolomics

In the first step, spectra were selected from the raw data for each polarity and the retention times of the chromatographic peaks were aligned. Unknown compounds with spectral peak intensity values over 10,000 were then detected and spectra. In the second step, missing values were filled with Compound Discoverer 3.3 and compounds were identified by different types of databases. Mzcloud was used to annotate compounds on MS/MS at tolerances within 10 ppm. Chemspider contains the BioCyc, the Human Metabolome Database (HMDB), and the Kyoto Encyclopedia of Genes and Genomes (KEGG) databases for precise mass-based annotation of features with a mass tolerance of 5 ppm and an S/N threshold of 1.5. In the third step, SERRF QC correlation was applied to reduce batch effects in all samples. In the fourth step, raw data from blank samples were used to label background compounds and the peak area of the samples/blanks was set to 5, enabling the use of background compounds to filter the background signal. Finally, a list of detected compounds, including exact molecular mass-to-charge ratio, retention time, compound name, and peak area, is exported in Excel format in QC normalization mode.

The exported compound names were manually checked before using MetaboAnalyst as a target list for pathway enrichemnt analysis and random forest analysis, both of which are online data processing tools. In addition, peak area lists were uploaded into SIMCA-P 14.1 software (Umetrics, Malmo, Sweden) for PCA analysis to assess stability of data between samples. A rank-sum test was performed on all metabolites detected by Compound Discoverer 3.3 to screen for metabolites that differed in expression levels between groups. Pathway enrichment analysis based on KEGG database was performed for the differential metabolites screened between groups using the pathway analysis module on MetaboAnalyst website.

### Proteomics analysis

In this study, the iST sample pretreatment kit (PreOmics, Germany) was taken for protein pretreatment. 2 µl of plasma samples were taken and 50 µl of lysate was added, centrifuged at 1000 rpm for 10 min at 95°C and then left to cool down to room temperature, trypsin digestion buffer was added, and the reaction was incubated with oscillation for 2 h at 37°C, 500 rpm, and the reaction was terminated by adding 100 μl of buffer. The peptides were desalted using the iST cartridge in the kit, eluted twice with 100 μl of elution buffer, then vacuum-dried and stored at -80 degrees Celsius.

The samples were analyzed by LC-MS/MS using an AUR3-15075C18 analytical column (15 cm*75 μm, 1.7 μm) with a gradient of 30 min, a column temperature of 50°C, and a column flow rate of 400 nl/min. The B-phase consisted of 80% acetonitrile with 0.1% formic acid, and the gradient was started from 4% of B-phase, increased to 28% within 15 min, and to 44% within the next 4 min. 4 min to 44%, and then to 90% in the next 4 min, maintained for 3 min and then equilibrated at 4% for 4 min. The mass spectrometer was set to diaPASEF mode with a scanning range of 349-1229 m/z and an isolation window width of 40 Da. During the PASEF MSMS scan, the collision energy increased linearly with ion mobility from 59 eV (1/K0 = 1.6 Vs/cm^2^) to 20 eV (1/K0 = 0.6 Vs/cm^2^).

DIA data were analyzed using the Spectronaut18 default parameters, i.e., BGS Factory Settings (default), with a sequence database of uniprot-Homo_sapiens (version 2022, 20610 entries), set for Trypsin zymolysis, and fixed modifications of the search library parameters as: Carbamidomethylation (C) 57.02 and variable modification is: Oxidation (M) 15.99, whereas according to the iRT peptide software the retention time and mass window can be automatically corrected to automatically determine the ideal extraction window. The criteria for protein characterization were Precursor Threshold 1.0% FDR and Protein Threshold 1.0% FDR; while the Decoy database was generated using a mutated strategy, i.e., the sequence of a random number of amino acids was selected to be disrupted, and the number of disrupted amino acids should be greater than or equal to two and less than half of the total length of the peptide; Spectronaut was automatically corrected and the data were normalized using a local normalization strategy, while peptides less than 1.0% FDR were quantified using MaxLFQ to complete the proteome (13).

The quality control of mass spectrometry data included liquid phase system, mass spectrometry system, qualitative results, quantitative results, etc. The parameters of quality control included missed cut, specific enzyme cleavage, non-specific modification, half-peak width, distribution of ion out time, distribution of ion charge number, and mass axis shift. The samples of the beneficiary group were compared with those of the non-beneficiary group, and the mean of the relative quantitative values of each group of samples in the comparison sample pair was calculated for each white, and the ratio of the mean values of each group of samples in the comparison sample pair was the Fold Change (FC). The p-value was calculated by t-test to determine the significance of the difference, and FC>1.2 or FC<-0.83 and p<0.05 were used as the screening criteria for differential proteins. The protein quantification form was exported according to the library search software, and data preprocessing was required before screening for differential proteins. Data preprocessing includes contaminant library protein removal, missing value filtering and filling. The missing value filtering and filling adopt the global missing value rejection and filling strategy, which is, the samples were grouped according to the group, in each group of samples, the protein with more than 50% missing values was considered to have 0 expression in that group of samples, and the protein values with missing value less than 50% were filled with the very small value, and the proteins with the percentage of missing value more than 50% were filtered out when the percentage of missing value in all groups was more than 50%.

While statistical analysis such as differential proteins include Principal Component Analysis (PCA), Partial Least Squares Discriminant Analysis (PLS-DA), Hierarchical Cluster Analysis (HCA) and so on. A variety of bioinformatics analysis tools including GO analysis, KEGG analysis, and protein-protein interaction networks were applied in proteomics analysis.

### Statistical analysis

R 4.2.2 was used for data analysis, metabolomics and proteomics were screened for predictor variables using random forest and stepwise regression respectively, 29 differential metabolites and 23 differential proteins between the beneficiary and non-beneficiary groups were included in the independent variables available for screening, a randomized stratification method was adopted to divide the data into training and testing sets according to 7:3, based on the training set, logistic regression was used to respectively Based on the training set, logistic regression was used to construct clinical risks, metabolomics, proteomics, metabolomics and proteomics prediction models, and the ROC (Receiver Operating Characteristic) curve was used to evaluate the differentiation of the models, and the AUC (Area Under Curve) was used to evaluate the effectiveness of the models.

IPA (Integrate Pathway Analysis) software was used to analyze the pathway enrichment of 27 differential metabolites and 23 differential proteins simultaneously, and the corresponding dual-omics combined pathway maps were obtained.

## Results

### Patient characteristics

From October 2022 to October 2023, a total of 47 patients were enrolled in this study, including 42 male patients and 5 female patients. 35 patients were included in the benefit group while 12 were included in the no-benefit group. 30 of 35 patients belonging to benefit group were classified into stable disease (SD) group and the other 5 patients were classified into remission group which consisted of complete response (CR) group and partial response (PR) group. The no-benefit group consisted of progressive disease (PD) group and death group. The details of each group are shown in **Table 1**.

**Table 1:** The detailed information of participants.

	Groups	PR/CR	SD	PD/DEATH
Gender	Male	4	28	10
	Female	1	2	2
Age		68.2±7.36	66.87±7.13	66.33 ± 10.27
	40~49	0	0	1
	50~59	1	5	2
	60~69	2	13	4
	70~79	2	11	4
	80~89	0	1	1
BMI		22.69±1.52	23.12±5.14	23.89±4.27
Pathological type	Adenocarcinoma	0	11	5
	Squamous cell carcinoma	5	19	4
	Carcinoid	0	0	3
Stage	III	1	8	5
	IV	4	22	7
Smoking history	Yes	3	26	11
	No	2	4	1
ECOG score	0	3	11	3
	1	1	13	5
	2	1	6	4

### Metabolomics

A total of 998 metabolites identified after initial process using Compound Discoverer 3.3 were checked according to the HMDB database, Chemspider database, and KEGG database, and a total of 392 of these metabolites that were endogenous to the human body were manually identified. Subsequently, SIMCA- P 14.1 software was used to further analyze the cleaned data, and Principal Component Analysis (PCA) was applied to analyze the stability of the samples, according to the PCA-X in **Supplementary Figure 1**. As it was shown, the QC samples have good aggregation in both positive and negative modes which proved that the mass spectrometer has reached a good stability level during the metabolomics analysis.

**Figure 1:**
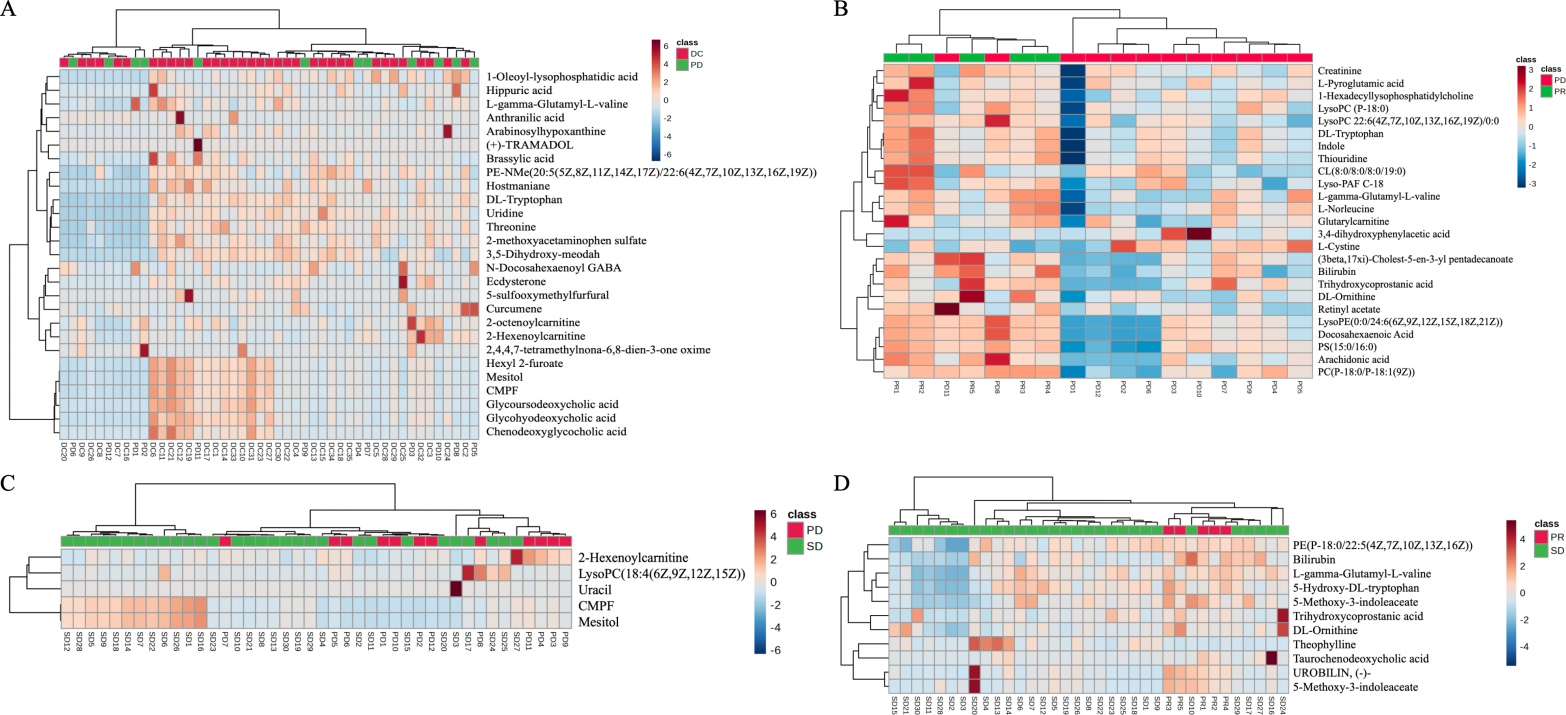
Heat map showing the results of the cluster analysis of differential metabolites between the progressed and mitigated groups **(A)**. Heat map showing the results of the cluster analysis of differential metabolites between the progressed and stabilized groups **(B)**. Heat map showing the results of the cluster analysis of differential metabolites between the mitigated and stabilized groups **(C)**. Heat map showing the results of the cluster analysis of differential metabolites between the benefited and non-benefited groups **(D)**.

Subsequently, the rank-sum test was performed on the peak values of each human endogenous metabolite for each sample in both the beneficiary and non-beneficiary groups, and 27 differential metabolites were obtained, which are shown in **Table 2**. The cluster analysis was also performed on the differential metabolites (**Figure 1A**).

**Table 2:** The differential metabolites between the beneficiary and non-beneficiary groups.

Name	Formula	m/z	RT(min)	Ion	FC	P
Uridine	C9 H12 N2 O6	279.03902	0.637	[M+Cl]-1	0.64432036	0.036
Threonine	C4H9NO3	119.05824	1.652	[M+H]+1	0.66352246	0.023
Mesitol	C9 H12 O	195.10212	6.412	[M-H+HAc]-1	0.39580597	0.026
Hostmaniane	C13 H18 O5	253.10824	5.78	[M-H]-1	0.57390195	0.028
Hippuric acid	C9 H9 N O3	178.05016	2.906	[M-H]-1	0.70977601	0.014
Hexyl 2-furoate	C11 H16 O3	195.10206	6.413	[M-H]-1	0.39580597	0.026
Ecdysterone	C27 H44 O7	481.31433	9.187	[M+H]+1	0.38236049	0.04
DL-Tryptophan	C11 H12 N2 O2	205.09714	2.404	[M+H]+1	0.66013304	0.048
CMPF	C12 H16 O5	239.0923	6.413	[M-H]-1	0.39684508	0.025
Brassylic acid	C13 H24 O4	243.16	6.988	[M-H]-1	0.71582303	0.034
Arabinosylhypoxanthine	C10 H12 N4 O5	267.07365	0.810	[M-H]-1	0.20327324	0.011
5-sulfooxymethylfurfural	C6 H6 O6 S	204.9807	1.703	[M-H]-1	0.04568696	0.001
2-methoxyacetaminophen sulfate	C9 H11 N O6 S	260.0236	0.808	[M-H]-1	0.58682834	0.022
1-Oleoyl-lysophosphatidic acid	C21 H41 O7 P	481.25793	9.683	[M+FA-H]-1	0.72535815	0.048
3,5-Dihydroxy-meodah	C22 H30 O7	405.1923	5.380	[M-H]-1	0.69134066	0.048
Glycoursodeoxycholic acid	C26H43NO5	516.29485	6.415	[M-H]-1	0.19795065	0.022
Glycohyodeoxycholic acid	C26H43NO5	516.97862	6.414	[M-H]-1	0.38797949	0.019
Chenodeoxyglycocholic acid	C26H43NO5	517.13943	6.414	[M-H]-1	0.45166525	0.045
PE-NMe(20:5(5Z,8Z,11Z,14Z,17Z)/22:6(4Z,7Z,10Z,13Z,16Z,19Z))	C48H74NO8P	823.51519	5.204	[M-H]-1	0.66109173	0.023
L-gamma-Glutamyl-L-valine	C10 H18 N2 O5	227.10327	2.131	[M-H-H2O]-1	0.78324428	0.048
Curcumene	C15 H22	203.17915	15.332	[M+H]+1	1.84164036	0.022
Anthranilic acid	C7 H7 N O2	138.05499	0.616	[M+H]+1	0.27665018	0.022
N-Docosahexaenoyl GABA	C26 H39 N O3	414.3001	11.948	[M+H]+1	1.72991327	0.02
2-Hexenoylcarnitine	C13 H23 N O4	258.17018	3.569	[M+H]+1	1.44288876	0.012
2-Octenoylcarnitine	C15 H27 N O4	286.20137	5.341	[M+H]+1	1.72271138	0.043
(3Z,6E)-N-Hydroxy-2,4,4,7-tetramethyl-6,8-nonadien-3-imine	C13 H23 N O	227.21174	4.983	[M+NH4]+1	3.62373447	0.025
(+)-TRAMADOL	C16 H25 N O2	264.1959	4.272	[M+H]+1	60.4912732	0.016

**Figure 2:**
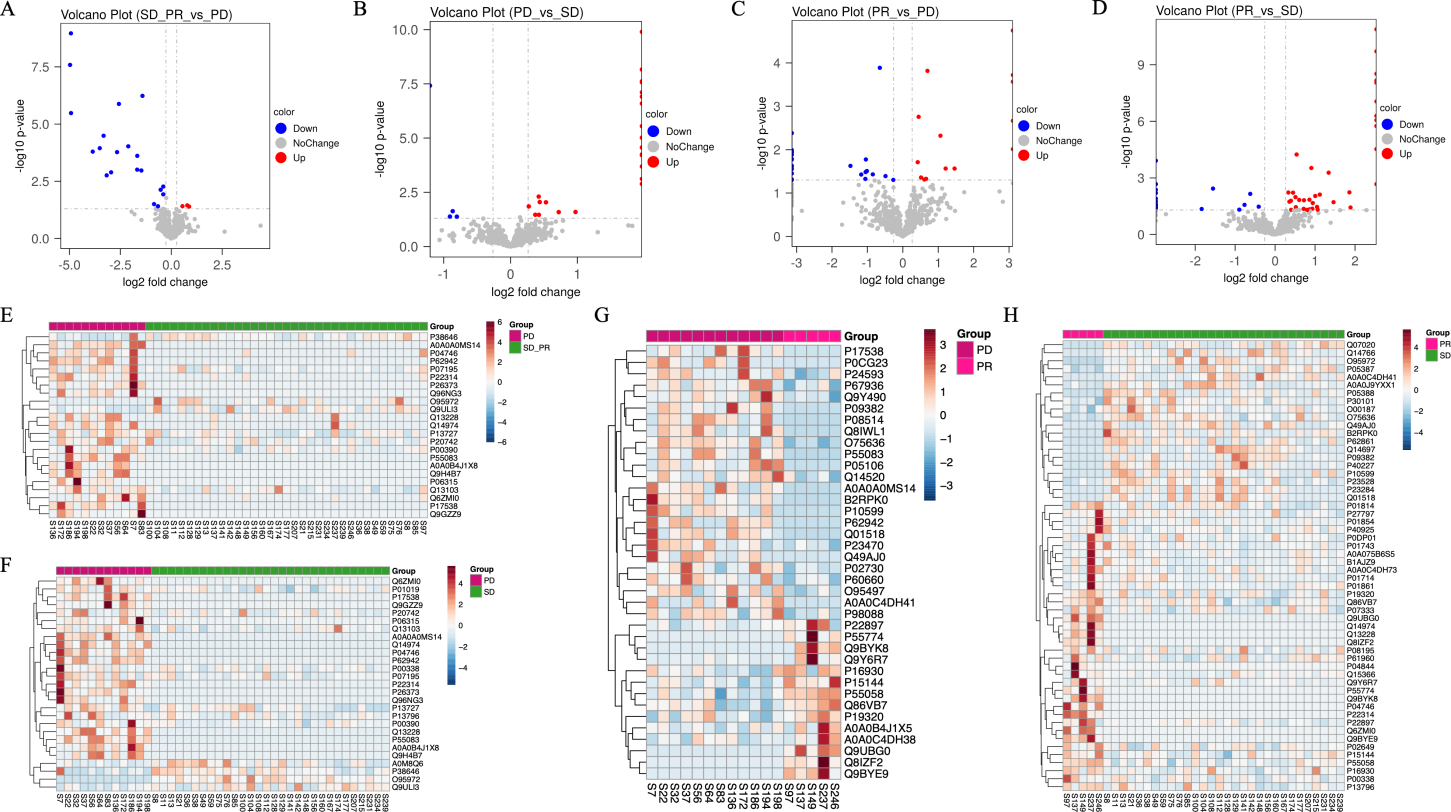
**(A)** Volcano plot of protein differences between the progressed and stabilized groups. **(B)** Volcano plot of protein differences between the relieved and progressed groups. **(C)** Volcano plot of protein differences between the relieved and stabilized groups. **(D)** Volcano plot of protein differences between the benefited and non-benefited groups. **(E)** Heat map showing the results of the cluster analysis of differential proteins between the beneficiary and non-beneficiary groups. **(F)** Heat map showing the results of the cluster analysis of differential proteins between the progressed and stabilized groups. **(G)** Heat map showing the results of the cluster analysis of differential proteins between the progressed and beneficiary groups. **(H)** Heat map showing the results of the cluster analysis of differential proteins between the mitigated and stabilized groups.

As it can also be seen in **Figure 2**, a two-by-two rank sum test comparison between the remission, stabilization, and progression groups revealed that there were 25 differential metabolites between the remission group and the progression group (**Figure 1B**), only 5 differential metabolites between the stabilization group and the progression group (**Figure 1C**), 11 human endogenous differential metabolites between the remission group and the stabilization group (**Figure 1D**).

Pathway enrichment analysis was performed on these 27 differential metabolites using the MetaboAnalyst website, and it was found that a total of 11 pathways were enriched (**Figure 2A**), among which were the metabolism of tryptophan and aminoacyl tRNA biosynthesis pathways had most differential metabolites on them, and this result was also verified in the subsequent IPA analysis. For some of the metabolites enriched on the relevant pathways we will validate this in the next section of our work.

**Figure 3:**
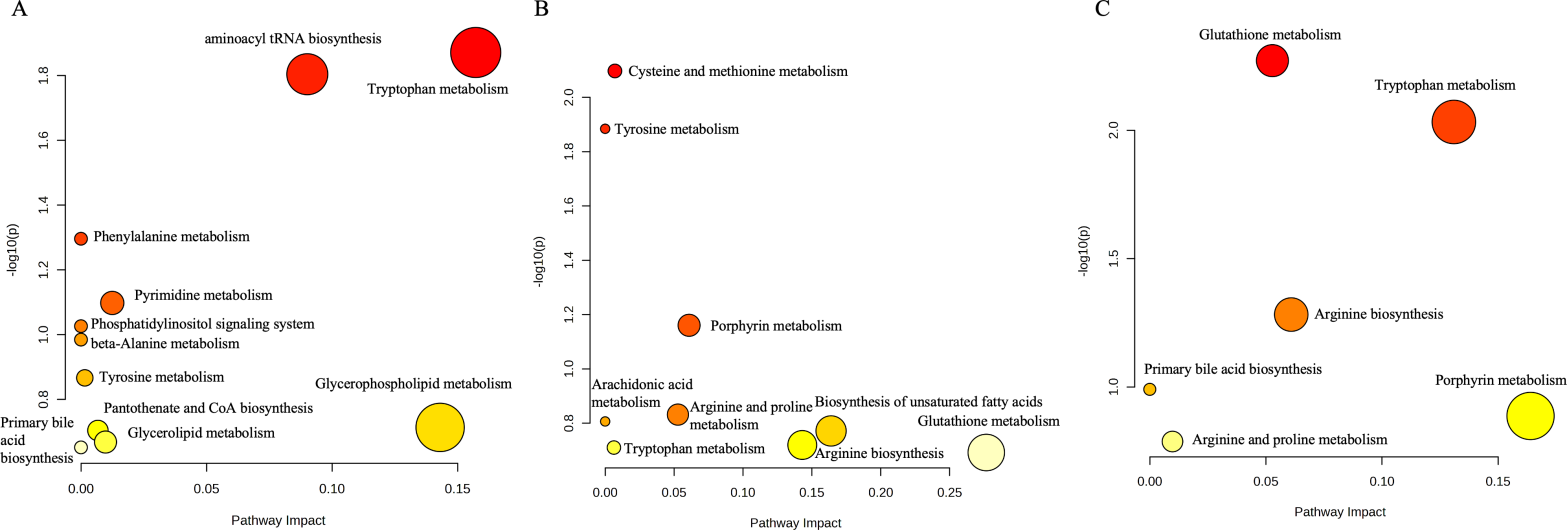
**(A)** Pathway enrichment results of differential metabolites between the beneficiary and non-beneficiary groups. **(B)** Pathway enrichment results of differential metabolites between the remission group and the stable group. **(C)** Pathway enrichment results of differential metabolites between the remission group and the progression group.

The three two-by-two comparisons had the highest number of differential metabolites between the PR and PD groups, and the differences in response to immune checkpoint inhibitor therapy for non-small cell lung cancer were also the greatest between these two groups of patients, with the highest number of pathways enriched. As shown in **Figure 3**, based on the pathway enrichment analysis of the three comparisons, it was found that the pathways enriched for differential metabolites between the remission group and the progression group were mainly glutathione metabolism and unsaturated fatty acid biosynthesis, whereas the metabolic pathways enriched for endogenous differential metabolites in the human body between the remission group and the stabilized group were mainly porphyrin metabolism and tryptophan metabolism. However, due to the lack of differential metabolites between stabilized group and progression group, corresponding pathway enrichment was not successful.

### Proteomics

**Figure 4:**
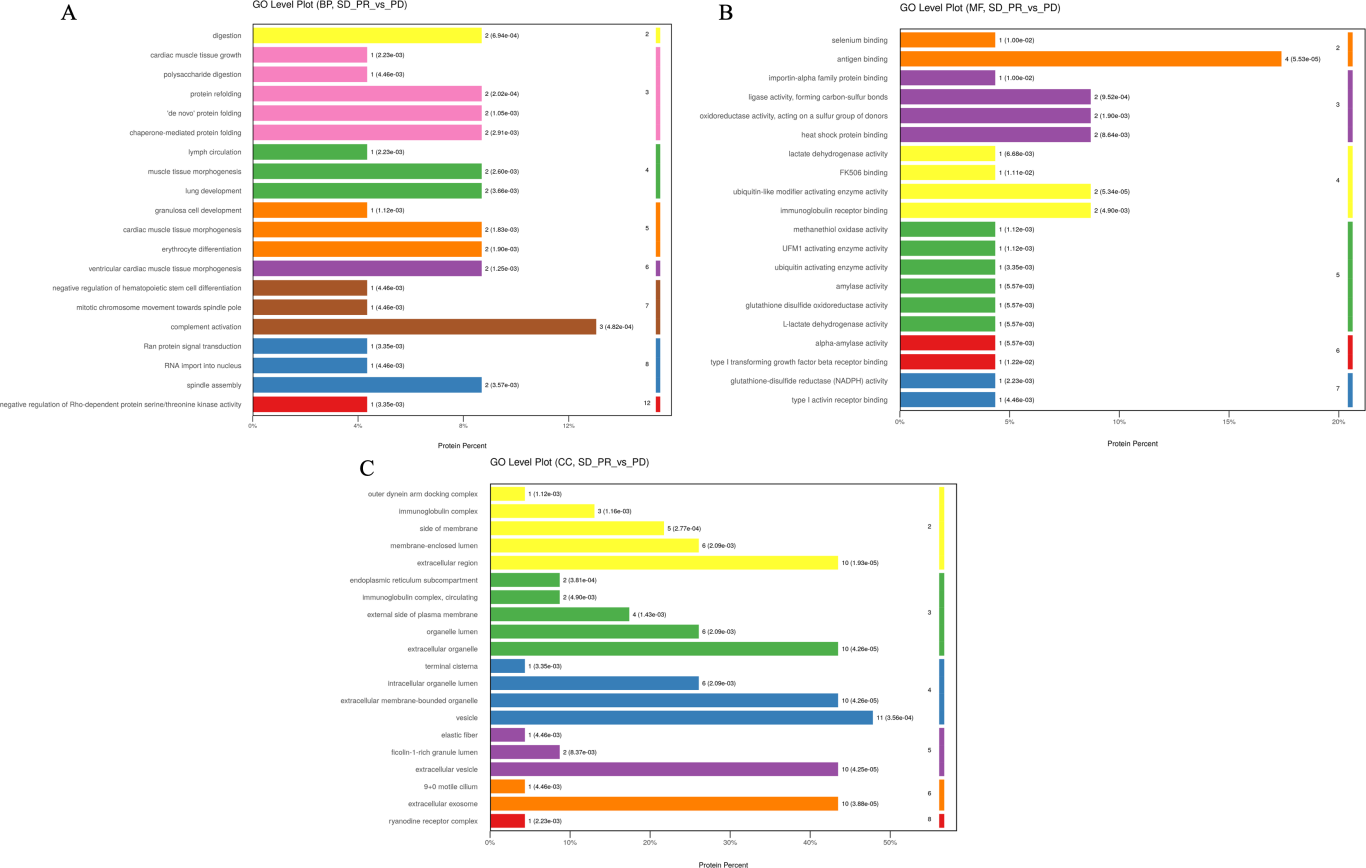
The top 20 entries with the smallest p-value under the three tertiary classifications of biological process **(A)**, molecular function **(B)**, and cellular components **(C)** in the GO analysis for differential proteins in the enrichment results under the three levels of classification and the GO level where they are located, and the horizontal coordinate is the percentage of differentiated proteins enriched by the entry.

Preliminary analysis of the proteomic mass spectrometry data was performed using Proteomics Discoverer software. 585 endogenous plasma proteins were identified in this study, and 23 proteins with statistically significant differences in expression levels between the beneficiary group and the non-beneficiary group, of which 20 proteins had higher expression levels in the non-beneficiary group, while only three proteins, namely GDF-9B (Growth/differentiation factor 9B), Stress-70 protein and Protein HEG homolog 1, had higher levels in the beneficiary group, respectively. GDF-9B, Stress-70 protein and Protein HEG homolog 1 were expressed at higher levels in the beneficiary group, which were respectively derived from BMP15 GDF9B, HSPA9 GRP75 HSPA9B mt-HSP70 and HEG1 KIAA237 gene translation as shown in **Table 3**. 27 differential proteins were identified between the progression group and the stabilization group, 56 differential proteins between the stabilization group and the remission group, and 38 differential proteins between the progression group and the remission group, and the results of the cluster analysis are shown in **Figure 4**.

**Table 3:** The differential proteins between the beneficiary and non-beneficiary groups.

Accession	Comparation	P.Value	Fold.ChangeRatio	Fold.Change	Entry Name	Protein names	Gene Names	Length	Mass
A0A0A0MS14	PR+SD VS PD	1.08652E-09	0.032800117	-30.487696	HV145_HUMAN	Immunoglobulin heavy variable 1-45	IGHV1-45	117	13508
A0A0B4J1X8	PR+SD VS PD	3.23403E-05	0.099875454	-10.01247	HV343_HUMAN	Immunoglobulin heavy variable 3-43	IGHV3-43	118	13077
O95972	PR+SD VS PD	0.038580205	1.46247003	1.46247003	BMP15_HUMAN	Bone morphogenetic protein 15, BMP-15	BMP15 GDF9B	392	45055
P00390	PR+SD VS PD	0.011719912	0.761090691	-1.3139039	GSHR_HUMAN	Glutathione reductase, mitochondrial, GR, GRase, EC 1.8.1.7	GSR GLUR GRD1	522	56257
P04746	PR+SD VS PD	0.000243714	0.313993078	-3.1847836	AMYP_HUMAN	Pancreatic alpha-amylase, PA, EC 3.2.1.1	AMY2A	511	57707
P06315	PR+SD VS PD	0.001279898	0.129021078	-7.7506716	KV502_HUMAN	Immunoglobulin kappa variable 5-2	IGKV5-2	115	12728
P07195	PR+SD VS PD	0.005344483	0.761768869	-1.3127341	LDHB_HUMAN	L-lactate dehydrogenase B chain, LDH-B, EC 1.1.1.27	LDHB	334	36638
P13727	PR+SD VS PD	0.038859222	0.63628859	-1.5716139	PRG2_HUMAN	Bone marrow proteoglycan, BMPG	PRG2 MBP	222	25206
P17538	PR+SD VS PD	1.31551E-06	0.168120627	-5.9481101	CTRB1_HUMAN	Chymotrypsinogen B, EC 3.4.21.1	CTRB1 CTRB	263	27713
P20742	PR+SD VS PD	0.031307466	0.556338526	-1.7974667	PZP_HUMAN	Pregnancy zone protein	PZP CPAMD6	1482	163863
P22314	PR+SD VS PD	9.34808E-05	0.231250165	-4.3243212	UBA1_HUMAN	Ubiquitin-like modifier-activating enzyme 1, EC 6.2.1.45	UBA1 A1S9T UBE1	1058	117849
P26373	PR+SD VS PD	0.000167921	0.157998345	-6.3291802	RL13_HUMAN	60S ribosomal protein L13	RPL13 BBC1 OK/SW-cl.46	211	24261
P38646	PR+SD VS PD	0.040103948	1.835046276	1.83504628	GRP75_HUMAN	Stress-70 protein, mitochondrial	HSPA9 GRP75 HSPA9B mt-HSP70	679	73680
P55083	PR+SD VS PD	5.84851E-07	0.374990958	-2.666731	MFAP4_HUMAN	Microfibril-associated glycoprotein 4	MFAP4	255	28648
P62942	PR+SD VS PD	2.59674E-08	0.031870013	-31.377458	FKB1A_HUMAN	Peptidyl-prolyl cis-trans isomerase FKBP1A, PPIase FKBP1A, EC 5.2.1.8	FKBP1A FKBP1 FKBP12	108	11951
Q13103	PR+SD VS PD	0.007390135	0.696348849	-1.4360618	SPP24_HUMAN	Secreted phosphoprotein 24, Spp-24	SPP2 SPP24	211	24338
Q13228	PR+SD VS PD	0.001065419	0.361369338	-2.7672519	SBP1_HUMAN	Methanethiol oxidase, MTO, EC 1.8.3.4	SELENBP1 SBP	472	52391
Q14974	PR+SD VS PD	0.000978067	0.313252529	-3.1923126	IMB1_HUMAN	Importin subunit beta-1	KPNB1 NTF97	876	97170
Q6ZMI0	PR+SD VS PD	0.000113049	0.087644349	-11.409749	PPR21_HUMAN	Protein phosphatase 1 regulatory subunit 21	PPP1R21 CCDC128 KLRAQ1	780	88314
Q96NG3	PR+SD VS PD	0.000159629	0.068986416	-14.495607	ODAD4_HUMAN	Outer dynein arm-docking complex subunit 4	ODAD4 TTC25	672	76655
Q9GZZ9	PR+SD VS PD	0.001735818	0.110825138	-9.0232236	UBA5_HUMAN	Ubiquitin-like modifier-activating enzyme 5, Ubiquitin-activating enzyme 5	UBA5 UBE1DC1	404	44863
Q9H4B7	PR+SD VS PD	3.30681E-06	0.032929714	-30.36771	TBB1_HUMAN	Tubulin beta-1 chain	TUBB1	451	50327
Q9ULI3	PR+SD VS PD	0.035527922	1.723700266	1.72370027	HEG1_HUMAN	Protein HEG homolog 1	HEG1 KIAA1237	1381	147461

PLS-DA analysis was performed on the detected proteins by subgroups to show the trend of aggregation or dispersion of the samples within and between groups. As shown in **Supplementary Figure 2** the PLS-DA analysis of the benefit group and the non-benefit group showed a better trend of separation between the two groups and a better trend of aggregation between the samples within the groups; if grouped according to the progression group, the stabilization group, and the remission group, the comparisons of any two of the three groups including the progression group versus the stabilization group, the stabilization group versus the remission group, and the progression group versus the remission group were included, and these comparisons were all in the PLS-DA analysis showed a better trend of intra-group aggregation and inter-group separation, while the PLS-DA between the three groups proved that the inter-group differences between the three groups were significant while the intra-group samples were highly aggregated.

Subsequently, a volcano plot was drawn for the detected proteins, which is a scatter plot taking the log2 value of the fold change as the horizontal axis and the -log10 value of the p-value as the vertical axis, and proteins with up-regulation of expression levels were labeled in red, proteins with no change in expression levels were labeled in gray, and proteins with down-regulation of expression levels were labeled in blue, and as can be seen in **Figure 3**, even though the expression levels of most detected proteins were not statistically changed in the comparison of the groups, there were some proteins that still demonstrated an up- or down-regulation trend in the comparison. In the comparison between the benefit group and the non-benefit group, only 3 differential proteins were expressed at higher levels in the benefit group, while 20 proteins showed a tendency to be expressed at lower levels in the benefit group. The hierarchical clustering analysis, in which the color shades are used to represent the magnitude of protein expression, is shown as a heat map, which again confirms the trend of expression levels of differential proteins in the comparison of groups in the volcano plot.

From the comparison between the PD group and the SD group, it can be seen that only 4 differential proteins showed a downward trend in the PD group while the remaining 23 differential proteins showed an upward trend in the PD group; whereas from the comparison between the PR group and the PD group, the difference in the number of differential proteins with upward and downward trends was small, with 24 differential proteins with a higher expression level in the PD group and 14 with a lower expression level in the PD group; the difference in expression level between the PR group and the progression group was small. In the comparison between the PR group and the SD group, there were 36 different proteins with increased expression levels in the PR group and 20 proteins with decreased expression levels in the SD group.

Subsequent bioinformatics analysis based on differential proteins included GO function analysis, KEGG pathway analysis with STRING-DB protein interaction network. Differential proteins in the beneficiary and non-beneficiary groups were firstly categorized at the first level, which included Biology Process (BP), Molecular Function (MF), Cellular Component (CC), and Complement Activation, Antigen Binding, and Vesicles were the three pathways or cellular components that accounted for the highest number of differential proteins in the relative categorization. Complement activation, the bioprocess with the highest percentage of enriched proteins, is located in the seventh level of classification, and three differential proteins are located in the pathway corresponding to this entry; antigen binding, the molecular function with the highest percentage of enriched proteins, is located in the second level of classification, and four differential proteins are located in this pathway, and the rest of the pathways that are enriched with more differential proteins and with lower p-values are immunoglobulin receptor binding, ligase. The other pathways with low p-values were immunoglobulin receptor binding, ligase activity to form carbon-sulfur bonds, and heat shock protein binding, all of which had 2 differential proteins enriched in the relevant pathway; whereas in the cellular components the structure of vesicles was enriched in 11 differential proteins, while the structures of extracellular vesicles, extracellular exocytosis and extracellular exocytosis were enriched in 10 proteins, which may be related to the fact that the samples came from peripheral blood plasma after centrifugation of removed hematopoietic cells (**Figure 4**).

**Figure 5:**
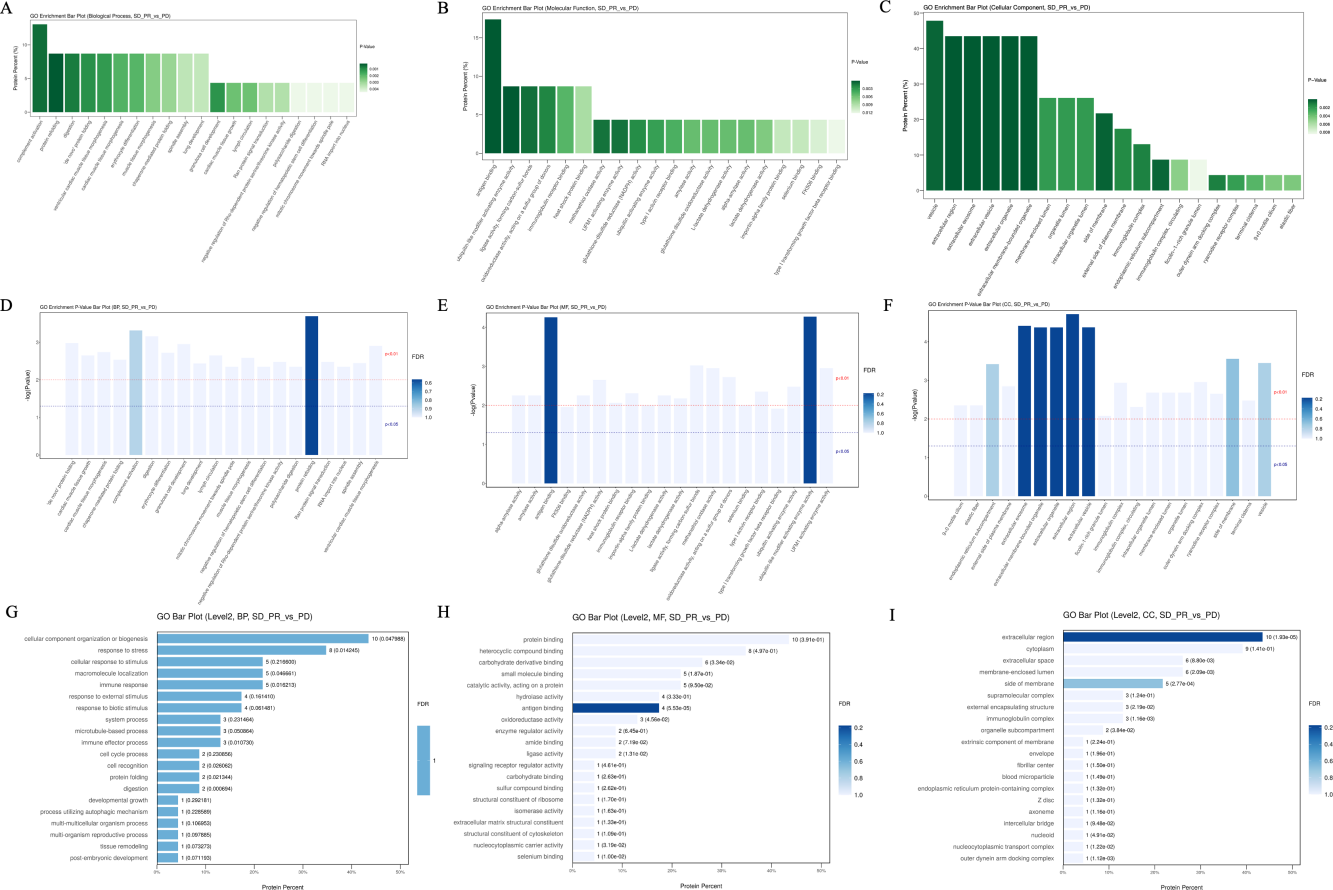
Bar graphs of GO enrichment. **(A–C)** P-values of the top 20 pathways with the most differential proteins enriched between the beneficiary and non-beneficiary groups. **(D–F)** FDR values of the top 20 pathways with the most differential proteins enriched under the three tertiary classifications. **(G–I)** Horizontal histograms of the top 20 tertiary or quaternary entries with the lowest p-value in the enrichment results under the lowest p-value secondary classification with the GO tier in which they were selected.

Under the primary classification of biological processes, the pathway of complement activation was enriched with the highest number of differentially differentiated proteins and the lowest p-value, with the highest confidence; the pathways with lower p-value and higher confidence also included protein recombination, *De novo* protein folding, erythrocyte differentiation, and lung development, which were also characterized by lower FDR values; and in the secondary classification of biological processes, cellular component organization/cellular component, CC, antigen binding, and vesicle were the pathways or cellular components that accounted for the highest number of differentiated proteins, respectively, in all three categories (**Figure 5**). In the secondary classification of biological processes, the pathway of cellular components, organization/biosynthesis was the pathway enriched to account for the largest number of differential proteins. Under the classification of molecular function, the antigen-binding pathway had a lower p-value and better FDR value, whereas protein-binding was the secondary classification under molecular function with the highest proportion of differentially enriched proteins, and ubiquitin-like modifier-activated enzyme activity was the pathway with the lowest p-value. Under the primary classification of cellular composition, vesicles were the cellular structure with the highest percentage of differentially differentiated proteins enriched to the structure, and the rest of the structures with a high percentage of differentially differentiated proteins were extracellular exosomes and extracellular regions, which also had the lowest p-value and the best FDR value under this classification. The top 20 entries with the lowest p-values in the enrichment results under the three classifications of biological processes, molecular functions, and cellular constituents were plotted against their GO tiers in a horizontal bar graph, with the horizontal coordinates of the bar graph being the percentage of differentially differentiated proteins enriched to that entry.

**Figure 6:**
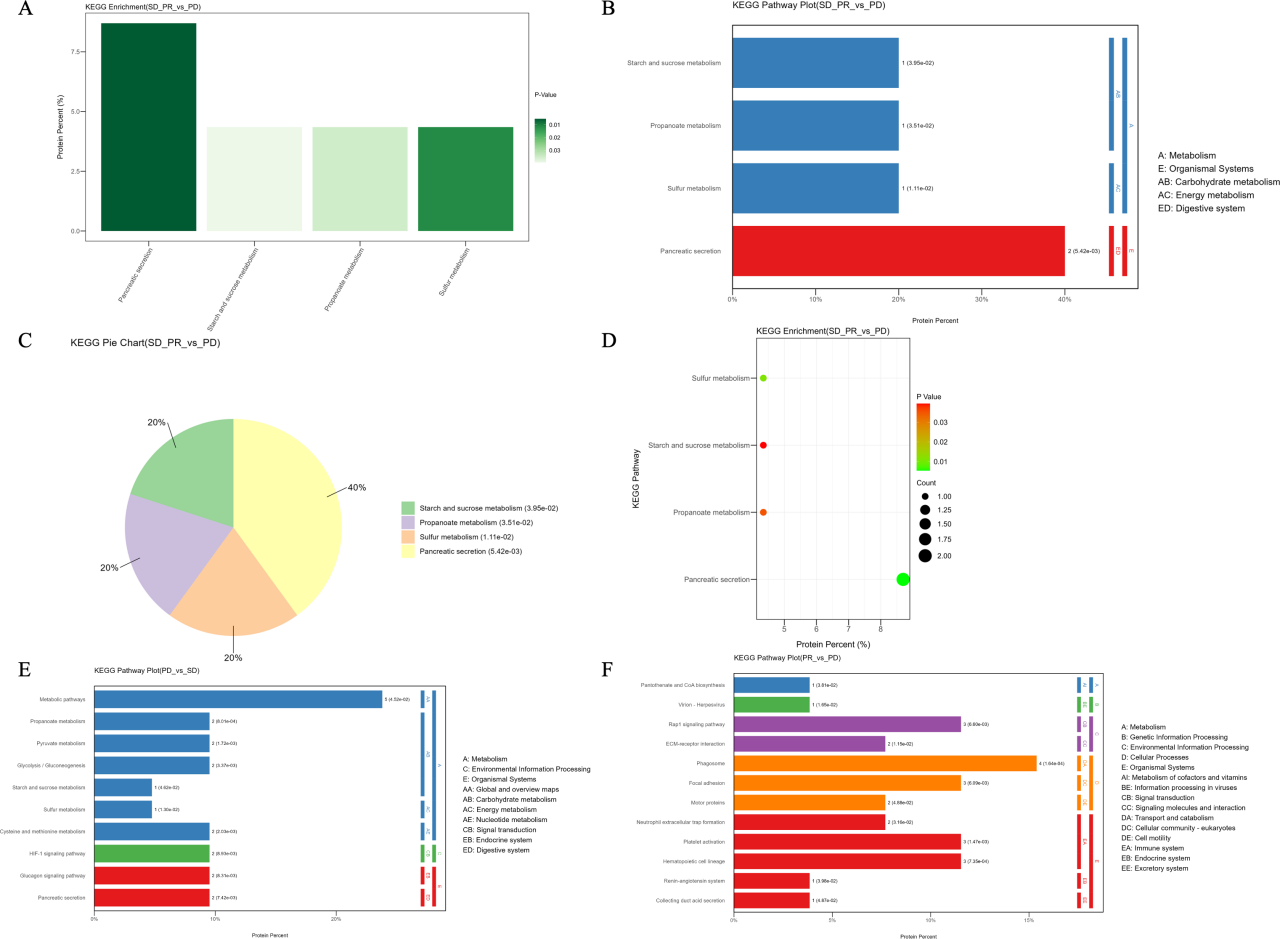
KEGG enrichment results. **(A)** The top 4 pathways with the smallest p-value, the vertical coordinates indicate the percentage of proteins in the pathway to the total number of differential proteins, and the darker the color indicates the smaller the p-value. **(B)** The vertical coordinates on the left show the specific metabolic pathways enriched, and the vertical coordinates on the right indicate the abbreviation of the primary and secondary taxonomic names. **(C)** P-value of the enriched metabolic pathways in order from the outside in. of the number of proteins enriched in the first 4 entries followed by a pie chart showing the percentage of the number of proteins in each entry. **(D)** Visualization of KEGG enrichment results in bubble charts, with the horizontal coordinates indicating the percentage of proteins in the pathway to the total number of differential proteins, and the color indicating the smaller p-value, and the size of the circle indicating the number of differential proteins in the pathway. **(E)** KEGG enrichment results of the differential proteins between the progression group and the stabilization group. **(F)** KEGG enrichment results of differential proteins between the remission and progression groups.

And as seen in **Figure 6**, the KEGG enrichment results were visualized in a similar way, the vertical coordinates of the front bar with the smallest p-value were counted to indicate the percentage of the proteins in the pathway to the number of differential proteins, and the darker the color, the smaller the p-value; whereas, the KEGG seven first-level macroclasses analyzed and their subordinate second-level classifications under which those pathways enriched by differential proteins belong to are labeled by means of the horizontal bar charts. Propionic acid metabolism, starch and sucrose metabolism belonged to the carbohydrate metabolism secondary classification under the metabolic classification, sulfur metabolism belonged to the energy metabolism secondary classification under the metabolic classification, and pancreatic secretion and other pathways belonged to the digestive system classification under the organic system; whereas pancreatic secretion and other pathways enriched in two differential proteins, most of the remaining multiplexes were enriched only in one differential protein, as the amount of proteins enriched by each pathway was relatively small, and therefore, the number of differential proteins enriched in each of these pathways was not large. The KEGG pathway analysis of differential proteins in the benefit group versus the non-benefit group was less reliable and had higher p-values for each pathway due to the small number of proteins enriched for each pathway. Whereas the entries with the highest number of differential proteins enriched in the comparison of the progression group with the stabilization group were metabolic pathways, the entries with the highest number of differential proteins enriched in the comparison of the remission group with the progression group were phagosomes.

**Figure 7:**
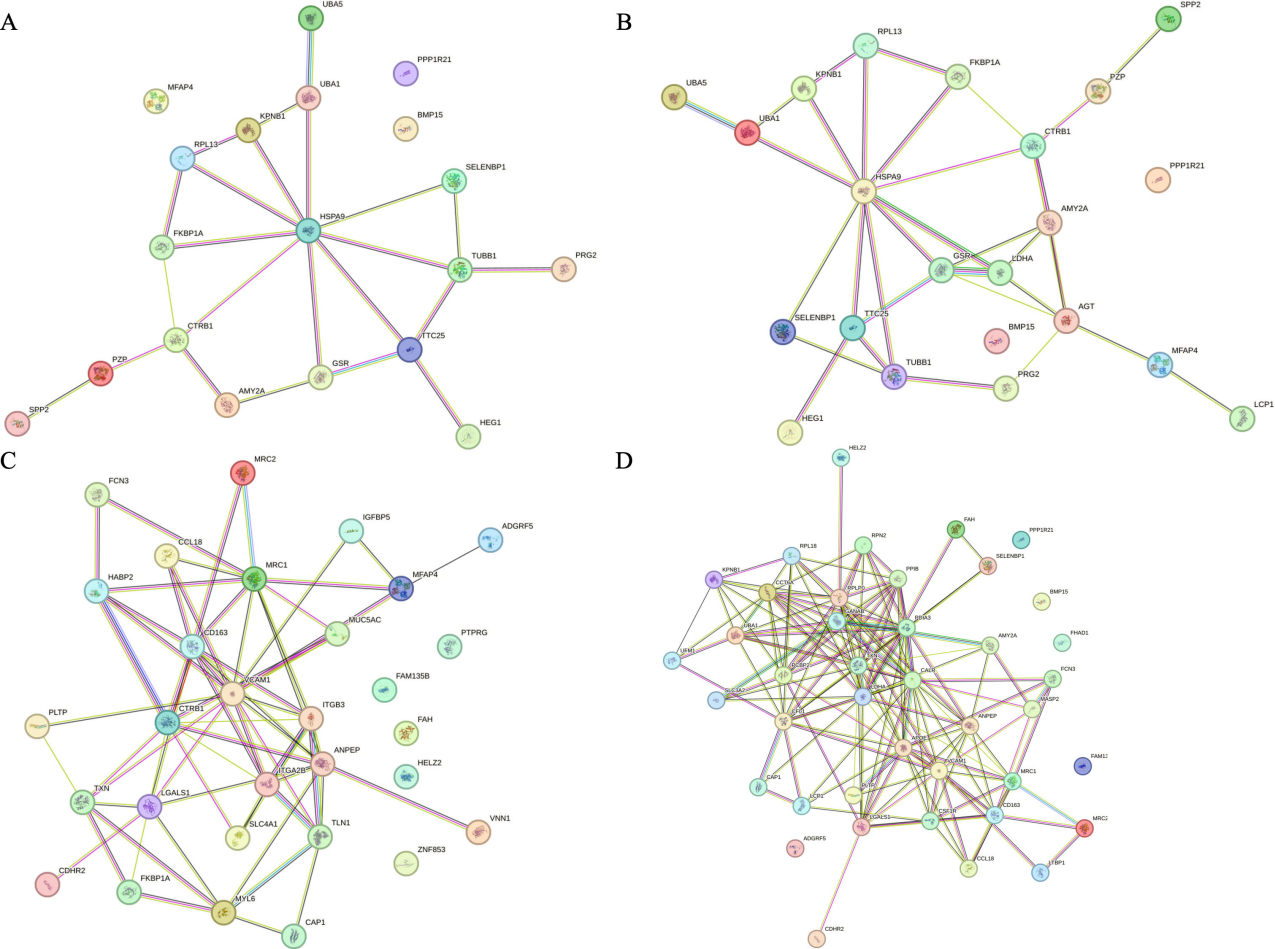
Results of String analysis of differential proteins. **(A)** Protein interaction network of differential proteins between the benefited and non-benefited groups. **(B)** Protein interaction network of differential proteins between the progressed and stabilized groups. **(C)** Protein interaction network of differential proteins between the relieved and progressed groups. **(D)** Protein interaction network of differential proteins between the relieved and stabilized groups.

The protein interaction analysis of differential proteins showed that, as shown in **Figure 7**, HSPA9 was the core of the interaction network composed of differential proteins, and the rest of the key nodes were CTRB1, TTC25, TUBB1, etc., which were closely related to the mutation of tumor driver genes, especially K-Ras mutation.

**Figure 8:**
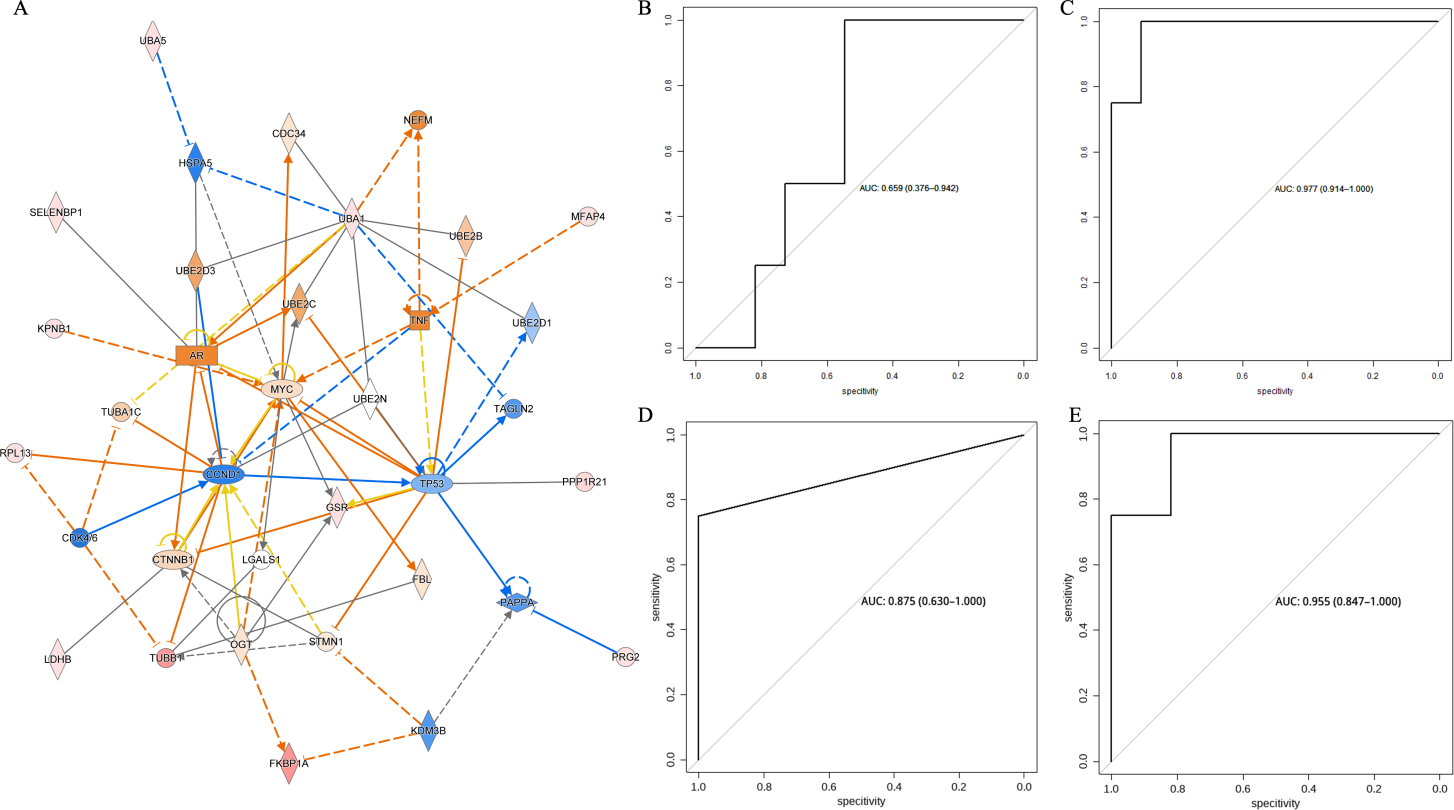
**(A)** IPA pathway enrichment analysis of differential metabolites and differential proteins between the beneficiary and non-beneficiary groups. **(B)** ROC curves in the test set based on predictive models constructed from biomarkers screened in metabolomics, proteomics and multi-omics. **(C)** ROC of prediction model based on clinical risks. **(D)** ROC of prediction model based on differential metabolites. **(E)** ROC of prediction model based on differential proteins. **(F)** ROC of prediction model based on both differential metabolites and proteins.

In contrast, as shown in **Figure 8A**, pathway enrichment analysis using IPA (Integration pathway analysis, QIAGEN, Dusseldorf, Germany) software showed that the expression levels of genes such as MYC, CCND1, and TP53 were important factors affecting the sensitivity of NSCLC to immune checkpoint inhibitor therapy.

### Multi-omics analysis

After screening by random forest and stepwise regression, metabolomics screened two differential metabolites, 5-sulfomethylfurfural and o-aminobenzoic acid, as biomarkers, while proteomics screened two differential proteins, Immunoglobulin heavy variable 1-45 and Microfibril-associated glycoprotein 4, as biomarkers. As shown in **Figures 8B–E** the early prediction model constructed based on two metabolic biomarkers, 5-sulfomethylfurfural and o-aminobenzoic acid, had an AUC of 0.977 in the test set while the early prediction model constructed based on two protein biomarkers, Immunoglobulin heavy variable 1-45 and Microfibril-associated glycoprotein 4, had an AUC of 0.875 in the test set. The early prediction model constructed from clinical factors had an AUC of 0.659 in the test set while the early prediction model constructed from dual omics biomarkers had an AUC of 0.955.

## Discussion

Tryptophan is an essential amino acid, and its metabolic pathway is the most metabolized and reliable metabolic pathway among the metabolic pathways enriched by differential metabolites in the beneficiary and non-beneficiary groups, while the metabolism of tryptophan is closely related to the treatment of immune checkpoint inhibitors in tumors, and its catabolic metabolism can reduce the body’s immune system whereas tryptophan itself can enhance the activity of CD8+ T-cells, increase CD8+ T-cells in tumor tissue and increase the infiltration of CD8+ T cells in tumor tissues, thus helping to induce apoptosis of tumor cells and slow down the growth of lung cancer (14). There are three metabolic pathways for tryptophan, including the indole pathway in the intestinal microbiota, the serotonin system pathway in intestinal chromaffin cells, and the kynurenine pathway in immune cells and the intestinal wall (15). All three pathways are relevant to immune checkpoint inhibitor therapy of tumors (16). Indole metabolites and kynurenine interact with the aryl hydrocarbon receptor to induce T regulatory cell differentiation, limit immune responses in Th17 cells, Th1 cells and produce anti-inflammatory mediators (17). Kynurenine leads to a decrease in CD8+ T cells infiltrating tumor tissue and mediates immune escape of tumor cells (18). Also, the serotonin system increases tumor cell proliferation and metastasis, while indole metabolites significantly reduce tumor growth (19).

Another pathway that was enriched by multiple metabolites in the comparison of the beneficiary and non-beneficiary groups is the biosynthesis of aminoacyl-tRNA. Aminoacyl-tRNA synthesis works by recognizing and catalyzing specific amino acid linkages to homologous tRNAs via aminoacyl-tRNA synthetases, which results in the precise matching of amino acids to tRNAs containing the corresponding paradigm codons, helping to achieve the accurate synthesis of proteins (20). Aminoacyl-tRNA synthetases are key enzymes in the translation process of mRNA, and the 20 essential amino acids correspond to 20 aminoacyl-tRNA synthetases, but there are fewer reports in the literature on the therapeutic effects of aminoacyl-tRNAs and anti-PD-L1/PD-1 therapy in tumors (21). Specifically, both threonine and tryptophan were enriched to aminoacyl-tRNA biosynthesis, and it is noteworthy that threonine was also an important differential metabolite between the beneficiary and non-beneficiary groups, and that serine/threonine is an important site in the WNT/β-catenin pathway during tumorigenesis, whereas the corresponding serine/threonine kinase plays an important role in this pathway (22). Transcriptomics revealed that serine/threonine kinase expression was negatively correlated with the expression of immune response markers in CD8+ T cells and the infiltration of dendritic cells, whereas further studies revealed that serine/threonine kinase expression levels were higher in patients with malignant tumors that were insensitive to anti-PD-L1/PD-1 therapy, and that genes associated with the WNT/β-catenin pathway and the MYC, a target gene of WNT, both had higher expression levels (23, 24). Knockdown of the serine/threonine kinase gene induced tumor shrinkage and increased immune cell infiltration in tumor tissues of malignant tumors ineffective for anti-PD-1 treatment in animal experiments, while synergistic effects of combining anti-PD-1 immune checkpoint inhibitors with anti-serine/threonine kinase targeting drugs could be observed in animal models, and synergistic effects were observed for the combination of Nivolumab and new therapy consisting of Nivolumab in combination with the dual anti-serine/threonine kinase inhibitor KPT-9274 is already undergoing clinical trials (25).

Immunotherapy for non-small cell lung cancer lung cancer has a close management with various protein pathways, and the activation of complement is the pathway with the most enriched differential proteins in the GO analysis of differential proteins between non-small cell lung cancer immunotherapy beneficiary and non-beneficiary groups, which plays an important role in immune evasion of tumors and activation of CD8+ T-cells, especially the complement factors produced by tumor cells could regulate tumor signaling and tumor tissue growth factors (26). For example, mutations in STK11 gene are thought to be associated with poor efficacy of immune checkpoint inhibitor therapy in NSCLC, while animal experiments have confirmed that the expression of complement pathway, including C3, is significantly increased in tumor cells with STK11 knockdown, and that complement C3 in NSCLC cell lines with STK11 knockdown can lead to a decrease in xenograft tumor growth in nude mice while tumor formation in mice with normal immune function basically disappeared. Mice with normal immune function, while population experiments have shown differential expression of complement C3 in non-small cell lung cancer patients with STK11 mutations (27). Whereas tumor cell-derived complement is now a possible target for immunotherapy in NSCLC, thus enhancing the anti-tumor capacity of the complement system, combined targeted blockade of C3aR (Component 3 Antibody Receptor)/C5aR (Component 5 Antibody Receptor) with PD-1/PD-L1 immune checkpoint inhibitor therapy appeared to have an antitumor synergistic effect (28).

Immunotherapy of non-small cell lung cancer lung cancer is also closely related to the expression level of the protein, HSPA9 as the core of the protein interaction analysis network of differential proteins, which is thought to be closely linked to the mutation of the tumor driver gene K-Ras, when there is a mutation in the K-Ras G12V gene depletion of HSPA9 can lead to the inhibition of the role of mitochondrial Ca2+ one-way transport protein is reduced and thus promote the death of normal fibroblasts (29). HSPA9, as a mitochondrial molecular chaperone, is often highly expressed and mislocalized in tumor cells with aberrant activation of MEK kinase and ERK kinase, and its depletion selectively kills tumor cells with high expression of B-Raf V600E or the chimeric protein ΔRaf-1:ER, and MEK-ERK-sensitive regulatory peptides in HSPA9 are not known to be active in the mitochondria (30). ERK-sensitive regulatory peptide binding domain in HSPA9 is important for cell survival or death. MEK-ERK increases mitochondrial permeability by promoting the interaction between adenine nucleotide translocase 3 (ANT3) and the peptidyl prolyl isomerase procyclic protein D (CypD), leading to cell death, whereas the depletion of HSPA9 leads to a reduction of its inhibitory effect on the MEK-ERK pathway and promotes cell death, affecting the cell’s ability to survive or die. of HSPA9 decreases thereby promoting cell death and affecting tumor prognosis.

CCND1 gene is one of the most central and associated pathways in the joint pathway analysis of metabolomics and proteomics, and it is thought to be associated with poor prognosis of lung cancer. For example, CCND1 and FGFR1 genes showed a tendency of co-expression in squamous lung cancer patients, and FGFR1 could promote the migration and invasion of squamous carcinoma cells by up-regulating the expression level of CCND1 gene to activate AKT/MAPK signaling to promote the process of epithelial mesenchymal transition, while knockdown of CCND1 gene in squamous lung cancer cell lines could significantly inhibit the proliferation of tumor cells, invasion and the process of epithelial mesenchymal transition (31). On the other hand, a study by a Korean scholar in 2023 found that malignant tumors with TP53 mutation and high expression of CCND1 gene on chromosome 11 tended to have a poor prognosis on treatment with immune checkpoint inhibitors, and TP53 was also one of the genes with a high number of associated pathways in the combined two-omics analysis of this study. As the most common oncogenic driver of lung adenocarcinoma, mutations in the TP53 gene promoted resistance to PD-1/PD-L1 inhibitors in a mouse lung adenocarcinoma model, and mutations in the TP53 gene have been shown to be associated with lower objective tumor remission rates after receiving Nivolumab in a large population-based cohort of lung adenocarcinomas (32). The MYC genes, on the other hand, have been closely associated with the function of T cells in the tumor microenvironment (24). Tumor cells with high expression of the MYC gene inhibit the JAK/STAT pathway thereby reducing IFN-γ stimulation and thus the efficacy of anti-PD-1/PD-L1 drugs. In contrast, inhibitors targeting MYC have been shown to inhibit tumor growth in mice, increase the infiltration of immune cells in the tumor microenvironment, and up-regulate the expression level of PD-L1 in tumor tissues making tumors more sensitive to anti-PD-1 immunotherapy (33).

The efficacy of the prediction model composed of differential metabolites was significantly higher than that of the prediction model constructed with biomarkers screened from differential proteomes, and even higher than that of the prediction model constructed by the combination of dual-omics, which may be attributed to the fact that the number of proteins that can be detected in the peripheral blood plasma is relatively small, and the total number of proteins detected is much lower than that of the total number of metabolites. The total number of proteins that can be detected is much lower than the total number of metabolites detected, so the biomarkers screened from peripheral blood plasma proteins may be less reproducible and less reliable than the biomarkers screened from peripheral blood plasma metabolites. Another aspect is that although proteins with more than 50% missing values have been excluded as differential proteins, differential proteins still have missing values in some samples, which may affect the modeling accuracy of the prediction models covering differential proteins.

In regard to the model constructed from clinical factors, there was no statistically significant difference in the clinical factors between the benefit and non-benefit groups. Among these factors, the relationship between CD8+ T cells and sensitivity to immunotherapy was the most significant. CD8+ T cells play a critical role in innate anti-tumor immunity, as their infiltration in tumor-bearing mouse models is associated with tumor size reduction and increased survival rates (34). However, with prolonged exposure to tumor antigens, CD8+ T cell function could deteriorate, showing a trend toward “exhaustion.” Immune checkpoint inhibitors can restore the function of CD8+ T cells, enabling them to once again target and eliminate tumor cells. Therefore, there is a close correlation between the absolute count of CD8+ T cells and the efficacy of immunotherapy. Additionally, patient age, BMI, and other cellular markers reflect the general health status of the patient, while tumor-related markers such as neuron-specific enolase, carcinoembryonic antigen, soluble fragments of cytokeratin 19, and squamous cell carcinoma antigen reflect the characteristics and status of non-small cell lung cancer (NSCLC). Although these clinical factors are associated with the efficacy of immunotherapy in advanced NSCLC, the model’s performance was modest, possibly due to the small sample size, the weak association between the included clinical factors (except for the absolute count of CD8+ T cells) and immunotherapy outcomes, and the fact that the absolute count of CD8+ T cells was measured in peripheral blood rather than directly in tumor tissue (35).

Although the metabolomics model, the proteomics model and the multi-omics model all showed good performance in the test set, due to the small total number of samples included in this study and the short period of time to follow up the NSCLC patients, the model still needs to be validated for model efficacy by long term follow up in external large scale population cohorts.

## Conclusions

Our study confirmed that metabolites and proteins in peripheral blood plasma can be used as biomarkers for predicting the immunotherapy sensitivity of unresectable stage III and IV NSCLC, and the early prediction models of NSCLC susceptibility to immune checkpoint inhibitors constructed by the combination of metabolomics, proteomics, and multi-omics showed good predictive performances, and CCND1, TP53, and MYC were three important genes affecting the susceptibility of NSCLC to immunotherapy.

## Data Availability

The data presented in the study are deposited in the iProX respository, accession number IPX0010564000.

## Supplementary material

Supplementary Figure 1
**(A)** PCA plots of the progressed, stabilized, and remission groups with QC samples in positive ion mode. **(B)** PCA plots of the progressed, stabilized, and remission groups with QC samples in negative ion mode.

Supplementary Figure 2
**(A)** Three-dimensional stereogram of PLS-DA for proteomics between the beneficiary and non-beneficiary groups. **(B)** Two-dimensional plot of PLS-DA for PLS-DA analysis between the progression, stabilization, and remission groups. **(C)** Two-dimensional plot of PLS-DA analysis between the two groups of the progression group and the stabilization group. **(D)** Two-dimensional plot of PLS-DA analysis between the two groups of the remission group and the progression group. **(E)** Two-dimensional plot of PLS-DA analysis between the remission group and the stabilization group.

## Glossary

ATP: Adenosine triphosphate
AUC: Area Under Curve
CR: Complete Response
DIA: Data Independent Acquisition
EDTA: Ethylene Diamine Tetraacetic Acid
FC: Fold Change
FDR: False Discovery Rate
HCA: Hierarchical Clustering Analysis
IDO1: indoleamine 2,3-dioxygenase 1
KEGG: Kyoto Encyclopedia of Genes and Genomes
LC-MS: Liquid Chromatography-Mass Spectrometry
NAD+: Nicotinamide adenine dinucleotide
PD-1: programmed cell death protein 1
PCA: Principal Component Analysis
PD-L1: Programmed cell death 1 ligand 1
PD: Progressive Disease
PLSDA: Partial Least Squares Discriminant Analysis
ppm: part per million
PR: Partial Response
QC: Quality Control
ROC: Receiver Operating Characteristic
rpm: revolutions per minute
SD: Stable Disease
SE: standard error
SNP: Single Nucleotide Polymorphism
TDO: Tryptophan-2,3-dioxygenase
TRC: Tumor repopulating cell
