# Supplementary figures and images for: Multi-omics analysis reveals the sensitivity of immunotherapy for unresectable non-small cell lung cancer
Source: Front Immunol. 2025 Feb 7;16:1479550. doi: 10.3389/fimmu.2025.1479550 (PMC11842339; doi:10.3389/fimmu.2025.1479550)

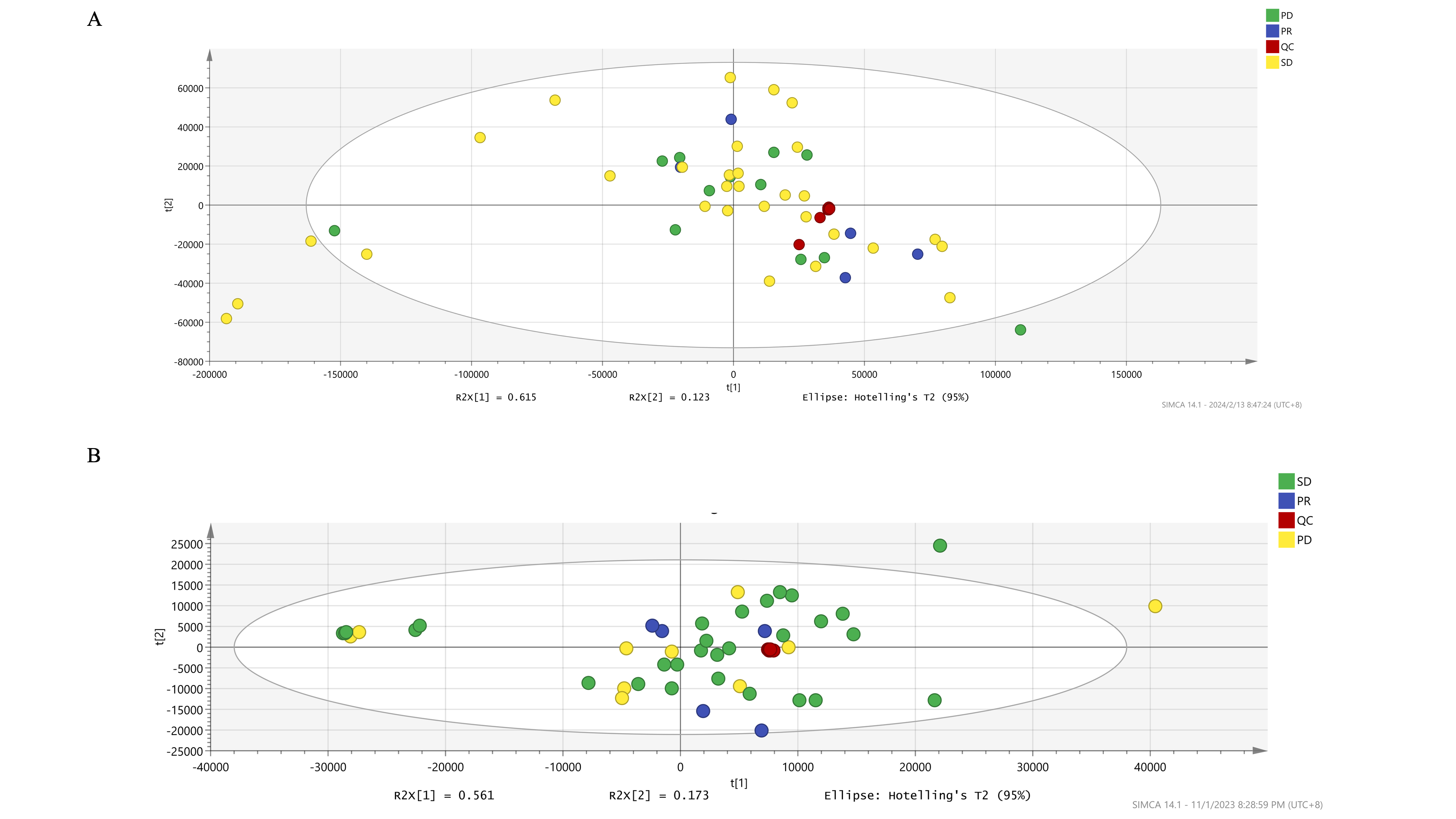

Supplement: Supplementary Figure 1 — (A) PCA plots of the progressed, stabilized, and remission groups with QC samples in positive ion mode. (B) PCA plots of the progressed, stabilized, and remission groups with QC samples in negative ion mode. [file Image1.tiff]

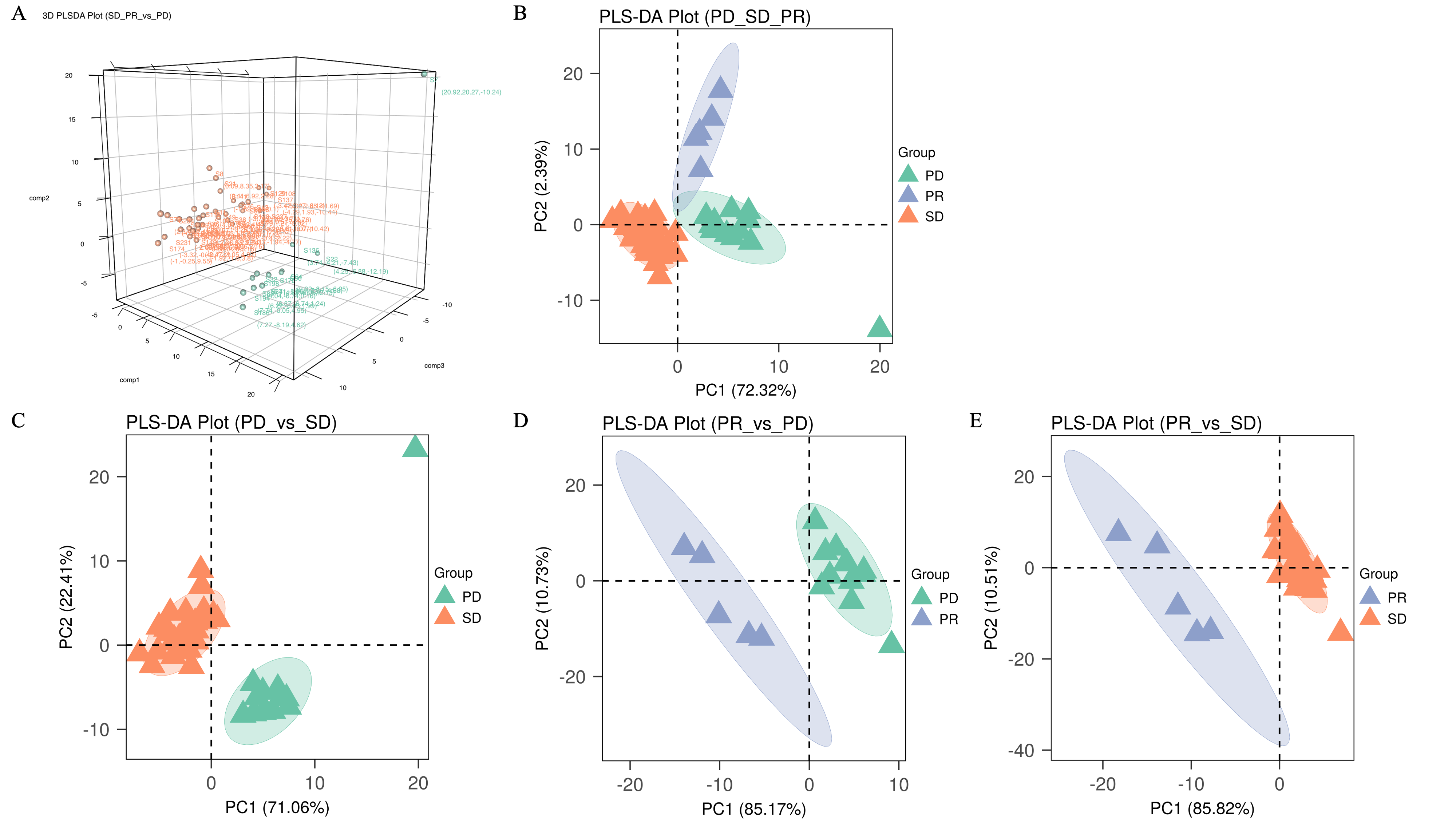

Supplement: Supplementary Figure 2 — (A) Three-dimensional stereogram of PLS-DA for proteomics between the beneficiary and non-beneficiary groups. (B) Two-dimensional plot of PLS-DA for PLS-DA analysis between the progression, stabilization, and remission groups. (C) Two-dimensional plot of PLS-DA analysis between the two groups of the progression group and the stabilization group. (D) Two-dimensional plot of PLS-DA analysis between the two groups of the remission group and the progression group. (E) Two-dimensional plot of PLS-DA analysis between the remission group and the stabilization group. [file Image2.tiff]
